# Enhanced CoAtNet based hybrid deep learning architecture for automated tuberculosis detection in human chest X-rays

**DOI:** 10.1186/s12880-025-01901-z

**Published:** 2025-09-26

**Authors:** Gunjan Siddharth, Ananya Ambekar, Naveenkumar Jayakumar

**Affiliations:** School of Computer Science and Engineering, Vellore Institute of Technology, Vellore, Tamil Nadu India

**Keywords:** Tuberculosis, CoAtNet, Image classification, Machine learning, Vision transformer, Deep learning, ICMR-NIRT

## Abstract

Tuberculosis (TB) is a serious infectious disease that remains a global health challenge. While chest X-rays (CXRs) are widely used for TB detection, manual interpretation can be subjective and time-consuming. Automated classification of CXRs into TB and non-TB cases can significantly support healthcare professionals in timely and accurate diagnosis. This paper introduces a hybrid deep learning approach for classifying CXR images. The solution is based on the CoAtNet framework, which combines the strengths of Convolutional Neural Networks (CNNs) and Vision Transformers (ViTs). The model is pre-trained on the large-scale ImageNet dataset to ensure robust generalization across diverse images. The evaluation is conducted on the IN-CXR tuberculosis dataset from ICMR-NIRT, which contains a comprehensive collection of CXR images of both normal and abnormal categories. The hybrid model achieves a binary classification accuracy of 86.39% and an ROC-AUC score of 93.79%, outperforming tested baseline models that rely exclusively on either CNNs or ViTs when trained on this dataset. Furthermore, the integration of Local Interpretable Model-agnostic Explanations (LIME) enhances the interpretability of the model’s predictions. This combination of reliable performance and transparent, interpretable results strengthens the model’s role in AI-driven medical imaging research. Code will be made available upon request.

## Introduction

Tuberculosis (TB) is an infectious disease caused by the bacillus mycobacterium tuberculosis, predominantly affecting the lungs. It spreads through the air when individuals with TB cough, sneeze or spit. TB occurs in all countries and affects people of all ages. In 2023, an estimated 10.8 million individuals fell ill with TB worldwide, including 6.0 million men, 3.6 million women and 1.3 million children [1]. The disease resulted in a total of 1.25 million deaths that same year. Five countries accounted for 56% of the worldwide total with India being the foremost contributor at 26%. TB is likely to have regained its status as the leading cause of death from a single infectious agent worldwide after being temporarily overtaken by coronavirus disease (COVID-19) for three years. Approximately a quarter of the global population is estimated to have been infected with TB. These alarming statistics underscore the urgent need for early and accurate diagnosis of TB to improve patient outcomes. Despite these challenges, TB is a preventable and treatable disease, offering hope for effective control and recovery.

Traditional diagnostic methods for TB include clinical assessments, physical examinations, laboratory tests, chest X-rays (CXRs), and computed tomography (CT) scans. Among these, CXRs are widely utilized due to their accessibility, cost-effectiveness, and ability to provide clear images of lung structures quickly. However, interpreting CXRs requires the expertise of highly skilled radiologists, who may not always be available, particularly in rural areas. This shortage of resources can result in many inaccurate or missed diagnoses. This highlights the necessity for computer-aided diagnostic (CAD) systems that can assist radiologists in enhancing the accuracy and efficiency of TB detection.

The rise of artificial intelligence (AI) in medical imaging offers promising solutions to these challenges. AI-driven models can serve as supplementary tools for healthcare professionals by aiding in the screening and treatment of patients. While AI cannot replace clinical diagnosis, it can significantly extend awareness and understanding of infectious lung diseases, contributing to the broader field of medical AI.

This study utilizes CoAtNet [2], a hybrid deep-learning architecture that combines the representational power of Convolutional Neural Networks (CNNs) with the global attention mechanisms of Vision Transformers (ViTs). While CoAtNet has demonstrated strong performance on general-purpose image classification tasks, its use in medical imaging, particularly for tuberculosis (TB) classification, is relatively novel. In this work, CoAtNet is adapted and fine-tuned on a CXR dataset, enabling it to learn domain-specific features to distinguish TB from non-TB cases. To further ensure transparency and clinical trust in model outputs, Local Interpretable Model-agnostic Explanation (LIME) is incorporated to identify the specific image regions that influence the classification. This helps contribute to the development of reliable and explainable AI tools in medical imaging. While the primary goal of this study is to achieve high-accuracy binary classification, it is important to situate this task within the broader evolution of medical AI. Recent advancements are pushing beyond simple diagnostic labels toward more comprehensive outputs, such as automated, paragraph-level medical report generation [3]. However, the success of these sophisticated systems is fundamentally dependent on the reliability of the underlying classification engine. An accurate, interpretable, and trustworthy diagnostic model, as developed in this study, serves as an essential prerequisite. By focusing on a robust and transparent classification of tuberculosis, this work lays a solid foundation upon which future, more complex diagnostic systems can be confidently built.

## Literature survey

### Convolutional neural networks

Numerous studies have shown that deep learning algorithms can effectively detect and classify lung diseases with high accuracy. CNNs have been extensively studied for TB detection due to their ability to extract hierarchical spatial features from CXRs. CNNs such as VGG-19 and EfficientNet, have been widely employed for this purpose [4]. These models demonstrate strong classification performance but often lack interpretability, [5] which is critical in clinical settings. To address this, segmentation-guided pipelines have been adopted, where lung regions are first extracted using models like UNET to focus the classifier on relevant anatomical structures [5, 6]. These help the CNN model to distinguish TB from normal cases with improved performance due to this pre-localization.

To enhance explainability, visual interpretation techniques like Gradient-Weighted Class Activation Mapping (Grad-CAM) have been widely used to highlight disease-relevant regions in CXRs [6]. Additionally, model-agnostic explanation methods such as LIME and SHapley Additive exPlanations (SHAP) have been applied to further improve the transparency of predictions. Recurrent structures like GRUs have also been integrated with CNNs to model spatial dependencies and improve robustness to anatomical variability, as seen in architectures like DCNN-GRU [7].

Despite their widespread adoption, CNN-based models face several limitations. First, CNNs rely heavily on local receptive fields, which restrict their ability to model long-range dependencies in images. This is particularly problematic for TB cases with diffused or scattered abnormalities appearing in CXRs. Second, many CNN-based approaches depend on preprocessing steps like lung segmentation, which can introduce errors and computational overhead when working with noisy or low-quality CXRs. These challenges highlight the need for more end-to-end architectures to enable more robust detection of complex patterns in CXRs.

### Vision transformers

Vision transformers have emerged as an alternative to CNNs for computer vision tasks, leveraging self-attention mechanisms to capture global contextual relationships within CXRs. Innovations like MaxViT [8] introduce multi-axis attention by linking convolutional features with transformer models. The block design sequentially combines local, global, and grid attention, improving long-range dependency modelling, while preserving sensitivity to fine-grained abnormalities like small cavitations. A study performed in [9], presents a ViT architecture trained on CXR images for TB classification. Attention-based visualization maps generated from Grad-CAM were reported as a tool for supporting clinical decision-making.

However, ViTs are not without limitations. They are computationally intensive and require substantial resources for training, making them less accessible in low-resource settings. They are prone to overfitting, especially when trained on limited datasets, due to their extensive parameterization and lack of strong inductive biases, which are found in CNNs. The overfitting issue is typically addressed by using large-scale pretrained datasets, such as ImageNet-21K, to achieve competitive performance.

### Hybrid architectures

To address the drawbacks of both CNNs and ViTs, hybrid architectures have been developed that leverage the strengths of both approaches. The authors in [7] introduced a newer hybrid DCNN-ViT-GRU model [10], which effectively combines the feature extraction capabilities of both CNNs and ViTs. Another model introduced in [11], integrated DenseNet121 with a multi-head self-attention mechanism to combine convolutional features with transformer-based global context modeling. In [12], a Double Attention Res-U-Net-based Deep Neural Network (DARUNDNN) was introduced, which combined residual CNNs with attention gates to classify TB and normal cases. The model further incorporated the Dingo Optimization Algorithm for enhanced feature selection, supported by preprocessing, segmentation, and feature extraction for improved accuracy. These studies demonstrate that augmenting CNNs with attention mechanisms can enhance the diagnosis of various thoracic diseases from chest X-rays.

Recognizing the strengths of hybrid architectures, the CoAtNet [2] model was developed by unifying convolution and self-attention mechanisms in a vertically stacked structure, achieving better generalization, capacity, and efficiency across various vision tasks. The stage-wise design allows it to model both fine-level patterns and larger anatomical structures, making it well-suited for medical imaging. CoAtNet has also shown strong performance on large-scale benchmarks such as ImageNet-21K, even when trained with less data, which is valuable in applications where labeled data is limited. The SynthEnsemble framework in [13] demonstrated CoAtNet’s clinical potential by working on the NIH ChestX-ray14 dataset. This work specifically leveraged CoAtNet’s ability to balance local feature extraction with global attention mechanisms, combining it with models like ConvNeXt V2 and Swin Transformer V2 through weighted averaging. CoAtNet has also been used in other medical applications such as pneumonia image classification [14], mammogram mass detection [15], and retinal disease diagnoses [16].

A two-step ResNet-ViT model presented in [17] is a dual-stream architecture where both backbones process the same input image and then perform feature fusion for multiclass classification of tuberculosis and pneumonia. The LungMaxViT model in [18] integrated CNNs with squeeze-excitation modules and a transformer backbone—a principle already embedded in CoAtNet’s design.

CoAtNet was chosen as the backbone in this study due to its ability to simultaneously improve generalization and model capacity within a unified architecture. Unlike other hybrid models like ResNet-ViT, CoAtNet transitions from convolutional to transformer blocks within a single cohesive network, reducing the need for manual feature fusion or separate training phases. Additionally, it supports ImageNet-pretrained variants, which help with transfer learning for medical tasks with limited data. It also offers lighter configurations, like CoAtNet-0 and CoAtNet-1, that have fewer parameters and computational demands, making it more suitable for clinical deployment compared to heavier architectures like MaxViT.

Hybrid models like CoAtNet are valuable in medical imaging tasks such as TB detection. While ViTs perform well when properly pre-trained, CNNs are easier to run in parallel, making them suitable and efficient for resource-constrained applications. By integrating the local feature extraction ability of CNNs and the global context understanding of ViTs, these models allow for context-aware feature selection, leading to a more complete view of disease patterns. To improve understanding of the model’s decisions, these models use XAI techniques offering visual insights into model predictions. LIME generates sharp, localized contours that highlight only the most relevant areas, aiding human interpretability by providing user-friendly explanations.

Having examined recent literature as summarized in Table 1, several recurring research gaps have been identified—most notably, inadequate handling of class imbalance, limited preprocessing strategies, and lack of interpretability methods. These challenges are directly addressed in this study through a comprehensively designed pipeline. This includes the application of preprocessing techniques such as oversampling, normalization, and extensive data augmentation to improve model generalizability. Additionally, ablation studies and comparisons with other state-of-the-art models are conducted to validate the performance improvements introduced.

**Table 1:** Summary of literature survey

Authors & Year of Publication	Dataset	Total number of images used	Data Preprocessing Techniques	Methods	Future Scope
Alshmrani et al. [4] (2023)	Lung CXR images from various sources	80,000 (Pneumonia, Lung Cancer, TB, Lung Opacity, COVID-19, Normal)	Image resizing, normalization	VGG19 + CNN	Identification of the region of interest during classification.
Nafisah & Muhammad [5] (2024)	Montgomery County, Shenzhen, Belarus CXR datasets	1,098 (692 TB, 406 Normal)	U-Net segmentation, image resizing, rotation augmentation	Xception, Inception-ResNet-V2, ResNeXt-50, MobileNet, EfficientNet, with XAI Grad-CAM	Addressing class imbalance and using preprocessing techniques like data augmentation.
Sharma et al. [6] (2024)	NIAID TB portal program dataset	1400 (700 TB, 700 control CXR)	U-Net segmentation, Image resizing, normalization	Xception CNN model with XAI Grad-CAM	Expanding data framework to larger and more recent datasets, use of more modern hybrid architectures.
Islam et al. [7] (2023)	IQ-OTH/NCCD lung cancer CT dataset, COVID-19-CT	IQ-OTH/NCCD lung cancer dataset: 2,073 CT scans; COVID-19-CT dataset: 7,593 COVID-19 images, 6,893 normal images, and 2,618 community-acquired pneumonia (CAP) images	z-score normalization, resizing, augmentations	CNN-GRU with XAI methods LIME, SHAP, and Grad-CAM	Use of larger and more diverse datasets. Exploring the impact of different data augmentation techniques or architectural variations on the model’s performance (Ablation studies)
Vanitha et al. [9] (2025)	Tuberculosis (TB) Chest X-ray Database on Kaggle	TB and normal CXR images	CLAHE, Gaussian blurring, resizing	Vision Transformer with XAI Grad-CAM	Comparisons with other advanced deep learning architectures, improve data augmentation techniques.
Islam et al. [10] (2024)	IQ-OTH/NCCD lung cancer CT dataset, custom merged datasets	2,073 CT scans from lung cancer dataset; 7593 COVID-19 images, 2618 CAP images, malignant lung cancer images, and normal images.	Image resizing, SMOTE to address class imbalance, Gaussian filtering, z-score normalization, data augmentation techniques	Hybrid (CNN + ViT + GRU) with XAI methods LIME and SHAP	Expanding the dataset used, explore more interpretability methods.
Singh [11] (2024)	4 CXR datasets: ChestX-ray14, CheXpert, MIMIC-CXR-JPG, IU-CXR	ChestX-ray14: 112,120 CXRs, CheXpert: 224,316 CXRs MIMIC-CXR-JPG: 377,110 CXRs IU-CXR: 7,470 CXRs, containing different disease categories	Image resizing, normalization, augmentation	Hybrid (DenseNet + transformer)	Addressing class imbalance, improve class labelling.
Balamurugan et al. [12] (2024)	Montgomery County, Shenzhen, and NIH Chest X-ray datasets.	Montgomery County dataset: 138 images (58 TB, 80 normal) Shenzhen dataset: 662 images (335 TB, 327 normal) NIH Chest X-ray dataset: 112,120 images	Resizing images, noise removal using Wiener filtering, segmentation using Adaptive Fuzzy C-Means (AFCM) clustering	Double Attention Res-UNet	Improving U-Net based segmentation, addressing inconsistent class labelling.
Ashraf et al. [13] (2023)	ChestX-ray14 dataset	112,120 CXR images containing 14 different disease categories	Image resizing, normalization, augmentation	SynthEnsemble (CNN + ViT + Hybrid) containing DenseNet, ConvNeXtV2, Swin V2, VOLO, CoAtNet, and MaxViT	Addressing class imbalance, improving accuracy, inclusion of interpretability methods.
Hadhoud et al. [17] (2024)	Publicly available Kaggle dataset	700 TB images, 3500 normal images	Image resizing, normalization, CLAHE, data augmentation, and oversampling for minority classes	Hybrid (ResNet-50 + ViT-b16)	Improving computational efficiency due to two stage architecture.
Fu et al. [18] (2025)	COVID-QU-Ex dataset, Chest X-ray14 dataset	2,352 COVID-19 cases, 2,265 non-COVID-19 cases, and 2,171 normal instances.	Image resizing, data augmentation, CLAHE, Gaussian filtering	Hybrid LungMaxVit (CNN + MaxViT)	Addressing challenges in imbalanced datasets and limited dataset size, and exploring medical foundation models.
Agrawal et al. [19] (2023)	Publicly available dataset sources	1125 CXR images (COVID-19, pneumonia, and no-findings)	Image resizing	CNN (Transfer Learning from ResNet50) with XAI methods LIME and Grad-CAM	Increasing dataset size.

This work contributes to the growing field of CAD, offering a practical solution for resource-constrained settings where radiological expertise may be scarce. The key contributions of this study are as follows:

1. A detailed preprocessing and augmentation pipeline addresses dataset noise and class imbalance.
2. An improved CoAtNet model is employed to enhance TB classification performance by combining the benefits of CNNs and ViTs.
3. Explainable AI is integrated through LIME, enabling localized, human-interpretable explanations of the model’s predictions to aid clinical decision-making.

The structure of this paper is organized as follows: section “METHODOLOGY” presents the Methodology, outlining the research methods employed, detailing the dataset used, preprocessing steps, the proposed model architecture, and XAI techniques. section “Results and evaluation” exhibits evaluation metrics and statistical analysis. The results are discussed with experimental outcomes and compared with existing approaches. Finally, section “CONCLUSION” concludes and summarizes the key findings and discusses their implications in future TB classification techniques.

## Methodology

This study employs a structured approach to classifying lung images into TB and non-TB categories. The three phases of the proposed framework include data preprocessing, model architecture modifications, and interpretability enhancements. Recognizing the medical implications of this task, precedence is given to methods that enhance model accuracy, transparency and reliability.

### Dataset and preprocessing

This research utilizes the IN-CXR tuberculosis dataset [20], sourced from the National Institute for Research in Tuberculosis (NIRT) under the Indian Council of Medical Research (ICMR) (https://www.nirt.res.in/html/xray.html). Human adult chest radiography across the geography of India was done as part of the National Tuberculosis Prevalence Survey [21] conducted during 2019–21. It comprises a large collection of DICOM X-ray images (postero anterior view), grouped as normal and abnormal categories.

The National TB Prevalence Survey represents one of the largest nationwide TB surveys globally and provides critical data widely used by major organizations including the World Health Organization (WHO) for national TB burden assessment and policy planning [22]. Numerous peer-reviewed systematic reviews and epidemiological studies have also cited this survey for TB risk factor analysis, infection prevalence and disease progression [23–25].

To the best of our knowledge, this study is among the pioneering efforts to utilize the IN-CXR dataset in its original form for developing a deep learning-based classification model for TB. The dataset’s application in computational TB detection remains limited. This research thus fills an important gap by exploring the potential of the IN-CXR dataset for AI-driven diagnostic support in tuberculosis.

**Fig. 1:**
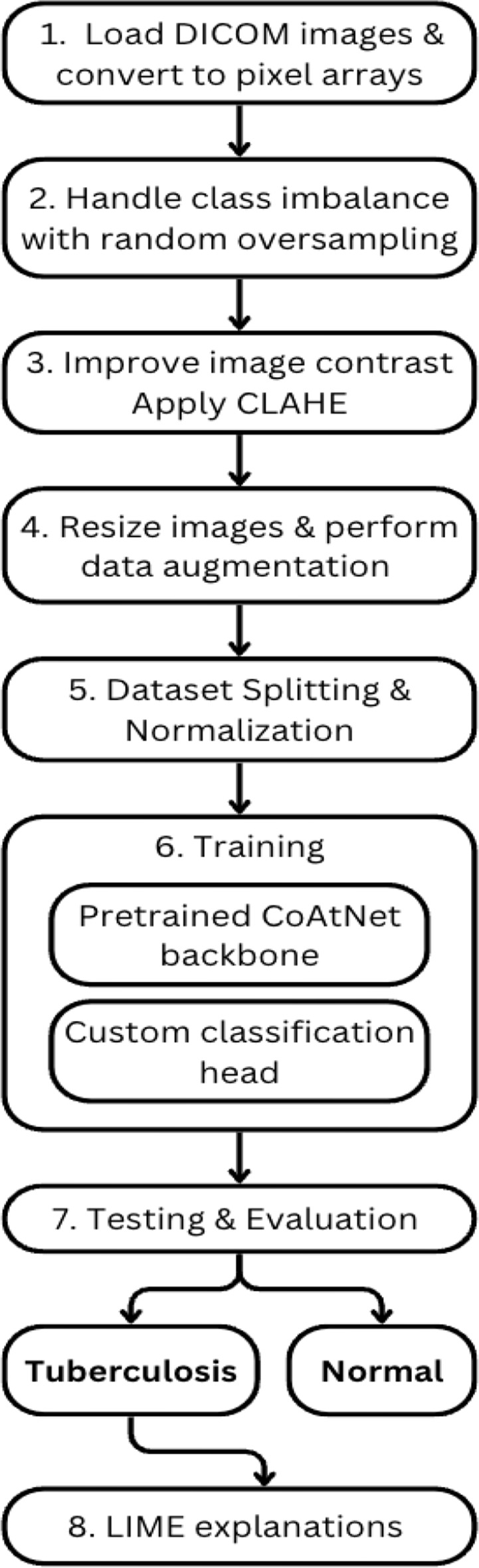
Oversampling of abnormal images

The dataset does not include detailed demographic information such as age and gender as it was anonymized. As such, the model’s performance might be influenced by these factors and may be biased towards the Indian demographic. The detailed processing steps are displayed in Fig. 1. The images are primarily stored in the DICOM format, which requires specialized software for visualization. To enable subsequent processing, pixel values are extracted from the DICOM image and normalized to an 8-bit (0–255) greyscale format. The images are then resized to a target size of 224 × 224pixels using area interpolation which is best for downscaling images as it prevents loss of details and reduces aliasing.

**Fig. 2:**
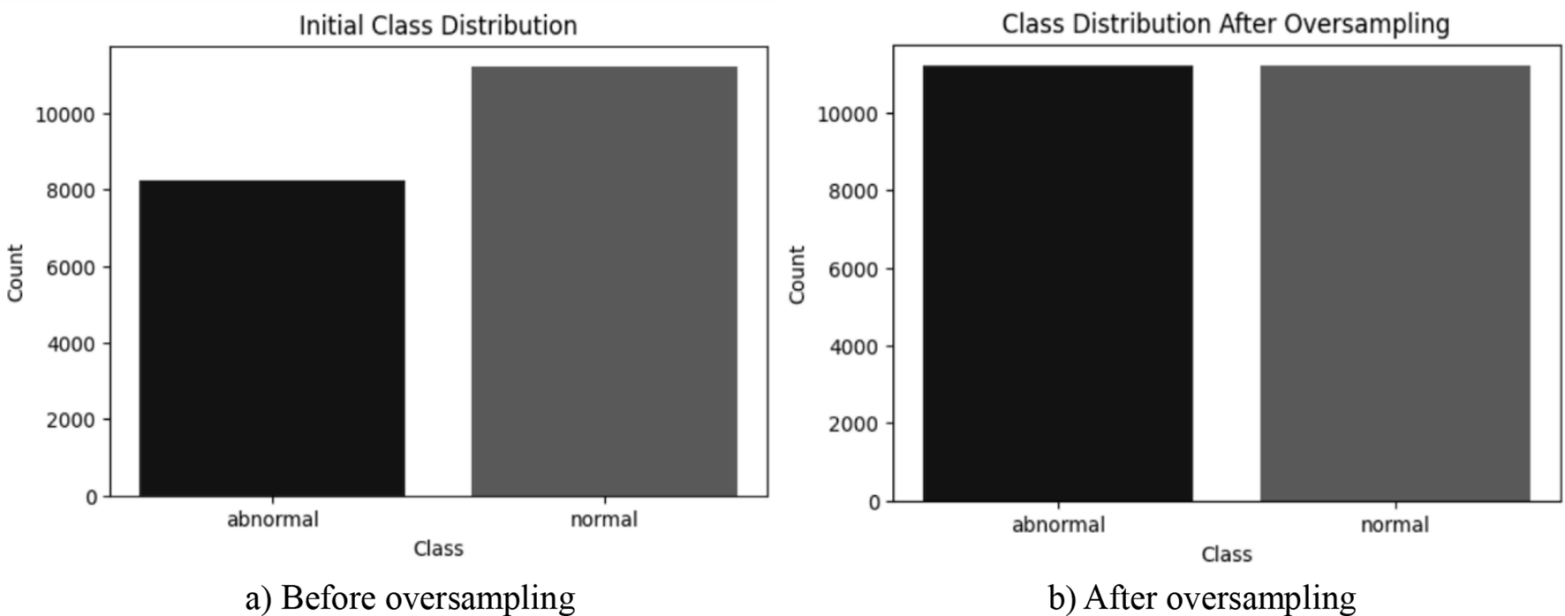
Processing pipeline

Furthermore, the dataset exhibits a class imbalance, with 11,203 normal cases and 8,232 abnormal cases. This imbalance presents a challenge, as it may lead to a model that does not generalize well across both classes. To address this issue, random oversampling is applied, which randomly duplicates images from the minority class to ensure a more balanced dataset. The final dataset consists of 11,203 normal and 11,203 abnormal images as depicted in Fig. 2.

To improve image contrast and enhance visibility of affected areas, the Contrast Limited Adaptive Histogram Equalization (CLAHE) technique is applied. It adjusts contrast by dividing images into small tiles and performing localized histogram equalization. This process mitigates variations in illumination across images, enhancing the clarity of critical medical features.

**Fig. 3:**
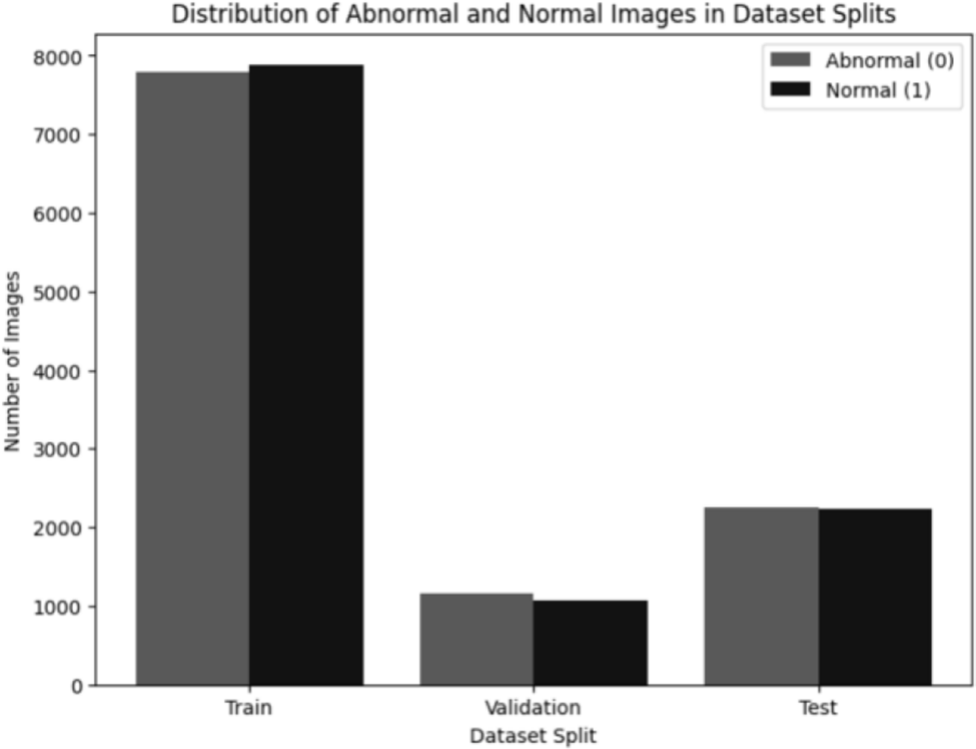
Class distribution of train, test and validation set

**Fig. 4:**
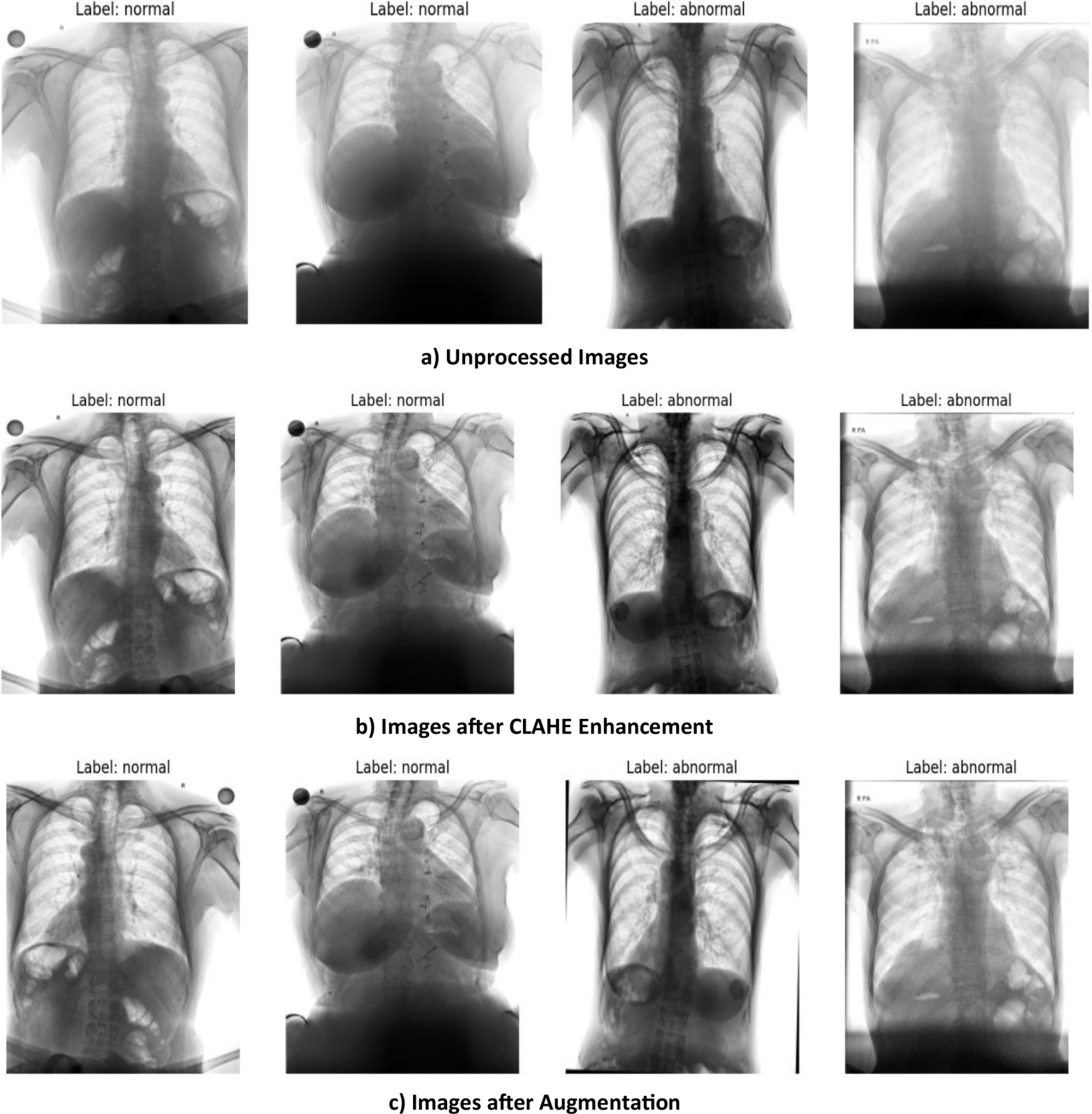
Sample images after each preprocessing step

The images are then augmented using random horizontal flips or random rotations between −5 and + 5 degrees with a probability of 50%. These augmentations introduce variations in the dataset, preventing overfitting and improving the model’s ability to generalize. The dataset is divided into training (70%), testing (20%), and validation (10%) sets. The class distribution in each set is illustrated in Fig. 3. The preprocessing pipeline is depicted in Fig. 4 with sample images at each step. The abnormal class represents TB patients and normal class represents non-TB patients.

### Model architecture

The backbone employed in this study is the CoAtNet architecture. As introduced by the authors in [2], it marries the inductive biases of convolutional neural networks with the global context modeling capabilities of transformer-based self-attention. This combination has proven highly effective across diverse data regimes in computer vision benchmarks. It integrates Mobile Inverted Bottleneck Convolution (MBConv) blocks [26], which are designed to capture fine spatial details, along with self-attention mechanisms to enhance global feature representation. The MBConv block, originally introduced for mobile efficiency in MobileNetV2, is built on an inverted residual structure and lightweight depthwise convolutions. Unlike traditional residual blocks, it first expands the channel dimension, then filters features, and finally reduces the channels allowing it to maintain a compact input and output representation while learning rich features internally. In the context of CoAtNet, this design allows the early convolutional stages to capture fine-grained local features from the CXR images with high computational efficiency, setting a strong foundation for the global context modeling performed by the subsequent transformer layers.

Several configurations of CoAtNet have been designed by integrating convolutional and transformer-based attention mechanisms, each tailored to optimize feature extraction at different stages. The architecture begins with Stage S0, a two-layer convolutional stem for initial feature extraction, followed by Stage S1, which employs MBConv blocks with squeeze-excitation to handle high spatial resolution efficiently. From Stage S2 onward, either MBConv (C) or Transformer (T) blocks can be used, with the constraint that convolutional stages must always precede transformer stages, as convolutions are more effective at capturing local patterns in early layers. This leads to 4 architectures with increasingly more transformer stages, C-C-C-C, C-C-C-T, C-C-T-T and C-T-T-T, where C and T denote Convolution and Transformer respectively. The C-C-T-T multi-stage architecture has been adopted, enabling a balanced feature representation.

Three models from the CoAtNet family have been explored—CoAtNet-0, CoAtNet-1, and CoAtNet-2—each differing in the number of blocks and depth of the network in each stage. CoAtNet-0 is the lightest, with fewer blocks and shallower depth, making it computationally efficient but less capable of handling complex patterns. CoAtNet-1 increases both the number of blocks and depth, improving its capacity to extract more intricate features. CoAtNet-2 adds more complexity with higher number of blocks and deeper layers, enabling it to handle large-scale datasets with greater feature extraction.

**Fig. 5:**
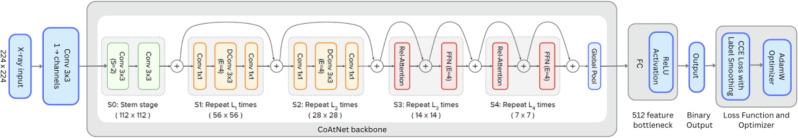
Model architecture

The proposed architecture as shown in Fig. 5 is an adaptation of the CoAtNet architecture, designed specifically for the classification of grayscale medical images. To align with the pre-trained CoAtNet framework, grayscale input images are transformed into three-channel RGB images using the Conv3x3 layer and resized to 224 × 224 pixels to match the input size of the model. Normalization is performed using standard ImageNet mean and standard deviation values to ensure consistency with the ImageNet pre-trained model.

At the final stage, architectural modifications are made to replace the default classification head of the CoAtNet model, which was originally trained to predict 1000 ImageNet classes. The original fully connected classification head is removed and replaced with a custom sequential layer. In this setup, the pretrained model’s output features are globally pooled, then passed through a 512-dimensional dense layer followed by a ReLU activation. The ReLU introduces non-linearity, helping the model learn more complex decision boundaries. Finally, a linear layer maps the features to a 2-dimensional output, predicting whether the input image belongs to the TB or non-TB category. Notably, this architecture can be easily adapted for multiclass classification tasks. If a suitable dataset with multiple class labels is available, the final output layer can be modified to include additional nodes, enabling the model to predict different disease categories.

### AdamW optimizer

To find optimal parameters for the model, the AdamW optimizer is applied. It is an extension of the Adaptive Moment Estimation (Adam) optimizer that incorporates weight decay for improved generalization. AdamW operates by maintaining an adaptive learning rate for each parameter while also applying decoupled weight decay regularization. The algorithm of the AdamW optimizer can be expressed by the following equations.

Calculating gradient:

1 $${g_t} \leftarrow {\nabla _\theta }f\left( {{\theta _{t - 1}}} \right)$$

Applying weight decay:

2 $${\theta _t} \leftarrow {\theta _{t - 1}} - \gamma \lambda {\theta _{t - 1}}$$

Updating biased first moment estimate:

3 $${m_t} \leftarrow {\beta _1}{m_{t - 1}} + \left( {1 - {\beta _1}} \right){g_t}$$

Updating biased second raw moment estimate:

4 $${v_t} \leftarrow {\beta _2}{v_{t - 1}} + \left( {1 - {\beta _2}} \right){g_t}^2.$$

Computing bias-corrected firstoment estimate:

5 $${\hat m_t} \leftarrow {{{m_t}} \over {\left( {1 - {\beta _1}^t} \right)}}$$

Computing bias-corrected second raw moment estimate:

6 $${\hat v_t} \leftarrow {{ {v_t}} \over {\left( {1 - {\beta _2}^t} \right)}}$$

Updating parameters:

7 $${\theta _t} \leftarrow \frac{{{\theta _t} - \gamma {{\hat m}_t}}}{{\left( {\sqrt {\widehat {{v_t}}} + \in } \right)}}$$

In the AdamW optimizer, *θ*_*t*_ represents the model parameters at step *t*, while *g*_*t*_ denotes the gradient of the loss function with respect to these parameters. The optimizer maintains two moment estimates: *m*_*t*_, the first moment estimate, which tracks the exponentially weighted moving average of past gradients, and *v*_*t*_, the second moment estimate, which tracks the moving average of squared gradients. The decay rates for these moment estimates are controlled by *β*_*1*_ and *β*_*2*_. Additionally, λ is the weight decay coefficient, which helps prevent overfitting and it has been set to 0.001. The learning rate, denoted as γ, determines the step size for parameter updates and has been initialized at 0.0001. Lastly, ϵ is a small constant added to the denominator to improve numerical stability and prevent division by zero.

### Categorical cross entropy (CCE) loss function

To enhance generalization, the model is trained using Cross-Entropy Loss with label smoothing, which prevents overconfidence in predictions. It is a widely used loss function for classification tasks which measures the difference between predicted probabilities and actual class labels. It is defined as

8 $$L\left( {y,\hat y} \right) = - \mathop \sum \limits_{i = 1}^C {y_i}log\left( {{{\hat y}_i}} \right)$$

where ${y_i}$ represents the true labels in a one-hot encoded format, ${\hat y_i}$ denotes the predicted probabilities, and C is the total number of classes. The logarithm ensures that incorrect predictions are penalized more heavily, guiding the model to adjust its weights for improved accuracy.

### Training and validation

The training process follows a structured pipeline where a forward pass is executed with mixed precision, followed by loss computation, backpropagation with scaled gradients, and optimizer updates. To ensure model reliability, checkpointing is implemented to save the best-performing model based on minimum validation loss. This prevents overfitting and ensures that only the most optimal model weights are retained for deployment. The model is trained for 50 epochs with a batch size of 32, a learning rate of 0.0001, and a weight decay of 0.001 to ensure stable learning and prevent overfitting. Label smoothing of 0.1 is applied to improve generalization by reducing overconfidence in predictions. The OneCycleLR scheduler with cosine annealing is employed with a warmup phase comprising 30% of the total training duration to optimize convergence. Automatic mixed precision with GradScaler is utilized to accelerate training and reduce memory consumption. A summary of the model layers is depicted in Table 2.

**Table 2:** Model layers, output shapes and parameters

Layer	Output Shape	Number of Parameters
Input	(1, 1, 224, 224)	0
RGB Repeat	(1, 3, 224, 224)	0
CoAtNet-1 Backbone	(1, 768)	40,922,982
Linear (768 → 512)	(1, 512)	393,728
ReLU Activation	(1, 512)	0
Linear (512 → 2)	(1, 2)	1,026

#### Experimental setup and reproducibility

All experiments are conducted on the Kaggle cloud environment with GPU acceleration using an NVIDIA Tesla P100 GPU (16 GB memory, CUDA 12.1). The execution environment provides access to 29 GB RAM and 57.6 GB of disk space. The implementation is performed in Python 3.10.12 using the PyTorch framework (v2.5.1) with Torchvision (v0.20.1) and the Timm library (v1.0.12) from HuggingFace. The IN-CXR dataset used in this study is publicly available, and the implementation code will be made available upon request to support reproducibility. The detailed training hyperparameters are summarized in Table 3.

**Table 3:** Hyperparameter values

Hyperparameter	Value
Optimizer	AdamW
Betas (AdamW)	(0.9, 0.999)
Epsilon (AdamW)	1e-8
Learning Rate	1e-4
Weight Decay	1e-3
Learning Rate Scheduler	Cosine Annealing
Batch Size	32
Number of Epochs	50
Loss Function	Cross-Entropy Loss
Label Smoothing Factor	0.1

#### K-Fold cross validation

**Fig. 6:**
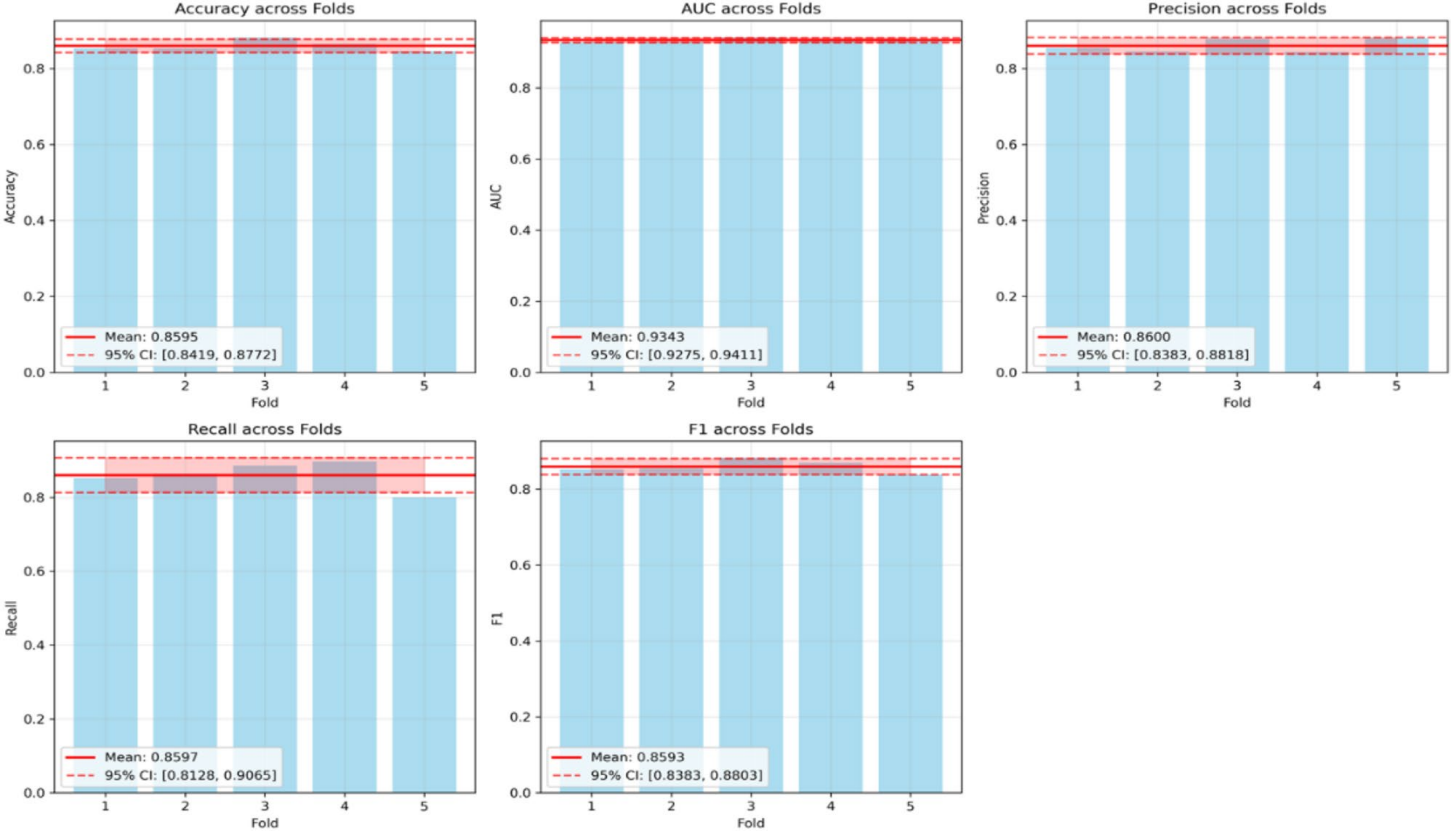
5-fold cross-validation results with 95% confidence intervals

To ensure robust model evaluation and mitigate the risk of overfitting to a specific train-validation split, we implement stratified k-fold cross-validation with k = 5 folds. This approach provides a comprehensive assessment of model performance by training and validating the model on different data partitions while maintaining the class distribution across all folds. Performance metrics including accuracy, AUC-ROC, precision, recall, and F1-score are computed for each fold, and statistical significance is evaluated by computing 95% confidence intervals using the t-distribution across the 5 folds to assess model consistency and generalization capability. Results are shown in Fig. 6.

The 5-fold cross-validation results demonstrate our model’s consistency and generalization capability. The model achieved a mean accuracy of 85.95% ± 1.42% with a narrow 95% confidence interval of [84.19%, 87.72%], indicating highly stable performance across all folds. The AUC-ROC score of 93.43% ± 0.55% demonstrates strong discriminative ability with minimal variance, while precision and recall metrics show balanced performance at 86.00% ± 1.75% and 85.97% ± 3.77% respectively.

The small standard deviations across all metrics (0.55% to 3.77%) confirm robust performance independent of specific train-validation splits. Importantly, the final model training results closely align with cross-validation findings, falling well within the established confidence intervals. This concordance validates the model’s generalization capability and confirms that the chosen architecture and hyperparameters are optimal for the dataset.

### LIME as an XAI technique

Explainable AI plays a crucial role in medical image classification, particularly in TB detection from CXRs. By enhancing transparency and accountability, XAI enables healthcare professionals to better understand and validate model predictions. This is essential for building trust in AI-driven diagnostic systems and supporting informed clinical decision-making.

To provide interpretability for our CoAtNet model’s predictions, we employ Local Interpretable Model-agnostic Explanations (LIME), which is a model-agnostic method that generates locally faithful explanations for individual predictions by approximating the decision boundary of complex models with a simpler, interpretable surrogate model. In the context of TB detection, LIME highlights which regions of an image contribute most significantly to the model’s classification outcome.

**Fig. 7:**
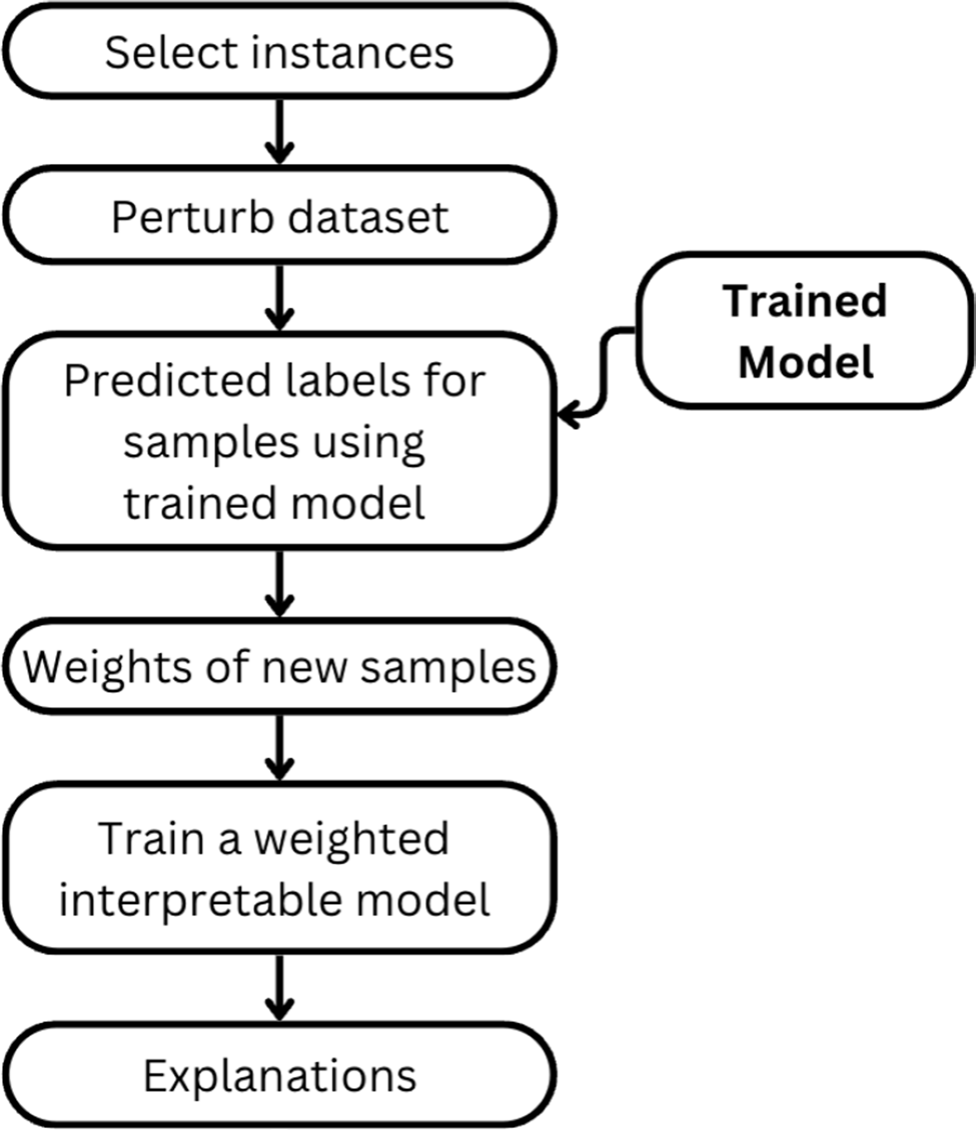
LIME process

**Fig. 8:**
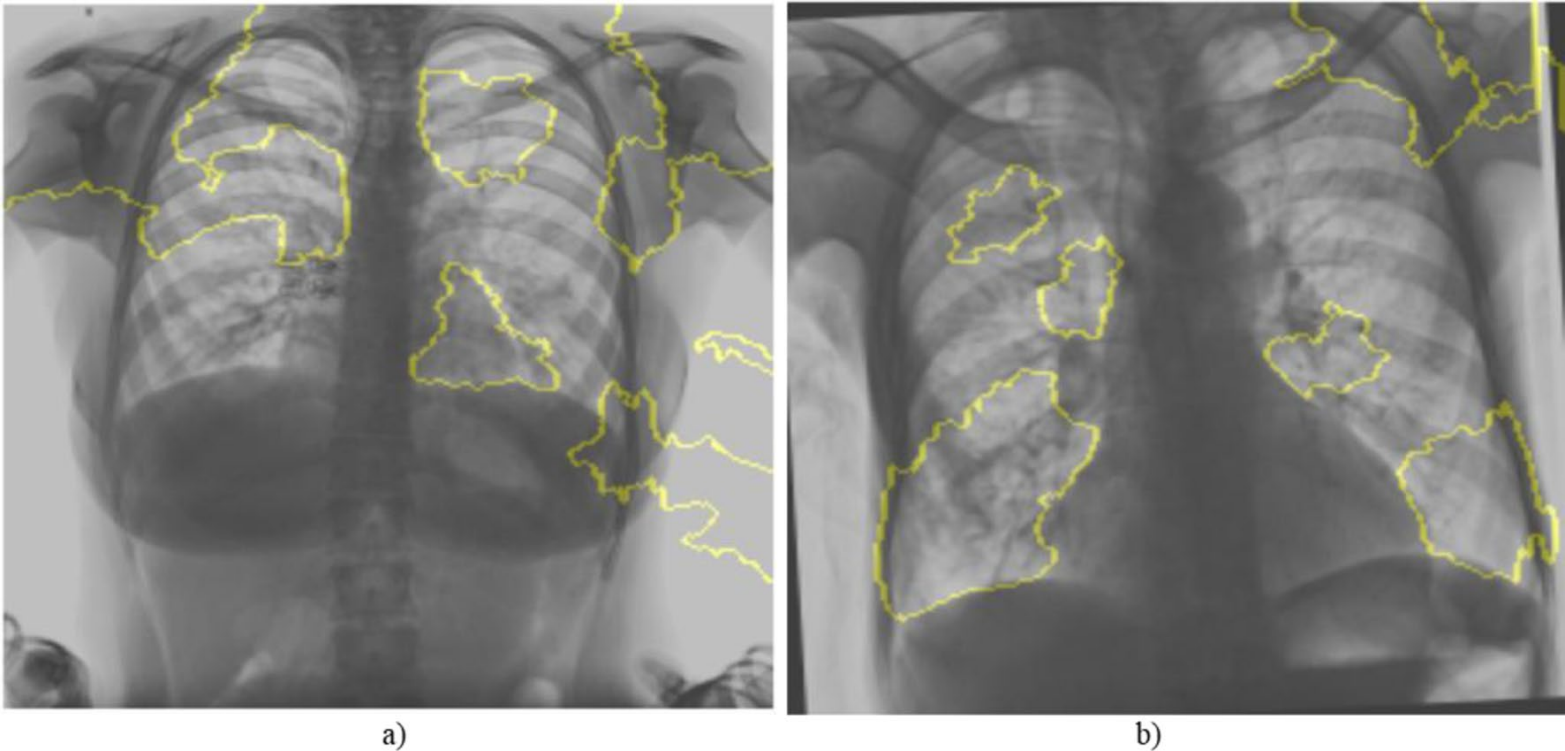
LIME explanation masks for TB-infected CXRs

As illustrated in Fig. 7, the LIME process involves segmenting the input image into superpixels, generating perturbed samples, training a local surrogate model, and producing a final explanation mask. Fig. 8 presents examples of LIME-generated masks on TB-positive images, visually indicating the radiographic features most influential in the model’s decision-making process. The steps are as follows:

- ⁠Image Segmentation: For the input CXR image we want to explain, LIME first segments it into a set of “superpixels”—contiguous patches of similar features. These superpixels serve as the interpretable components and fundamental units for perturbation.
- Perturbation: A new dataset is generated by creating numerous “perturbed” versions of the original image, where each version is created by randomly hiding a combination of superpixels.
- ⁠Prediction of Perturbations: The trained CoAtNet model predicts the probability of TB for each of these perturbed images.
- ⁠Weighting and Surrogate Model Training: The perturbed samples are weighted based on their similarity to the original image. A simple, interpretable model (e.g., weighted linear regression, typically a sparse model such as Ridge Regression) is then trained on this new dataset to learn a local decision boundary that mimics the CoAtNet model’s behaviour.
- Explanation Generation: The coefficients learned by the surrogate model indicate the importance of each superpixel. Superpixels with high positive coefficients, identified as strong evidence for a TB diagnosis, are visualized as a heatmap on the original image. The top K influential superpixels (K = 10) are visualized as a binary saliency mask overlaid on the original grayscale CXR image. This mask highlights the specific regions that contributed most significantly to the classification (e.g., TB-positive or TB-negative).

While LIME provides localized explanations by identifying influential superpixels, this study does not incorporate radiologist-annotated ground truth for lesion-level validation. Consequently, although the LIME-generated heatmaps highlight regions of interest that appear visually consistent with pathological features, their alignment with clinically validated TB lesions cannot be confirmed. Thus, these heatmaps should be seen as model-derived saliency maps, not definitive pathological indicators.

This limitation is consistent with gaps identified in existing literature, where prior studies employing LIME [2, 7, 10] and Grad-CAM [5, 9] have similarly provided region-level visual explanations without leveraging radiologist-annotated lesion masks. These works also relied on visual consistency rather than direct validation against clinical ground truth, underscoring a broader challenge in the field. Nevertheless, LIME addresses a significant aspect of model transparency by offering instance-specific insights, complementing performance-based evaluation metrics. Future studies could strengthen clinical validity by incorporating radiologist-annotated CXRs as a benchmark to assess whether the model’s attention aligns with established markers of tuberculosis. Such comparisons would enhance the model’s trustworthiness and support its potential integration into real-world diagnostic workflows.

## Results and evaluation

### Confusion matrix

The Confusion Matrix is computed, providing a detailed breakdown of the model’s performance across all classes on the test set. Table 4 reflects the principle of the confusion matrix that shows the number of true positives (TP), false positives (FP), true negatives (TN), and false negatives (FN), offering insight into where the model is making errors.

**Table 4:** The principle of confusion matrix

Confusion Matrix		Predicted Class	
		Positive	Negative
Actual Class	Positive	TP	FN
	Negative	FP	TN

**Fig. 9:**
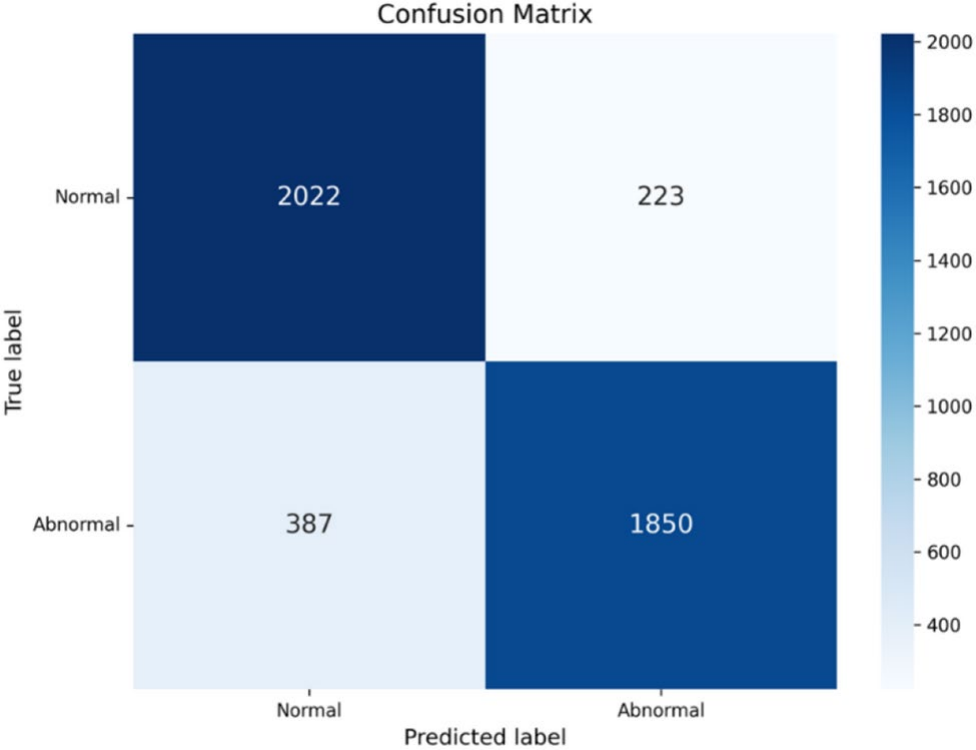
Confusion matrix

As shown in Fig. 9, the confusion matrix indicates that the model successfully identified 2,001 normal cases as true negatives and 1,868 abnormal cases as true positives. These results demonstrate the model’s ability to effectively distinguish between normal and abnormal classes.

### Model performance

**Fig. 10:**
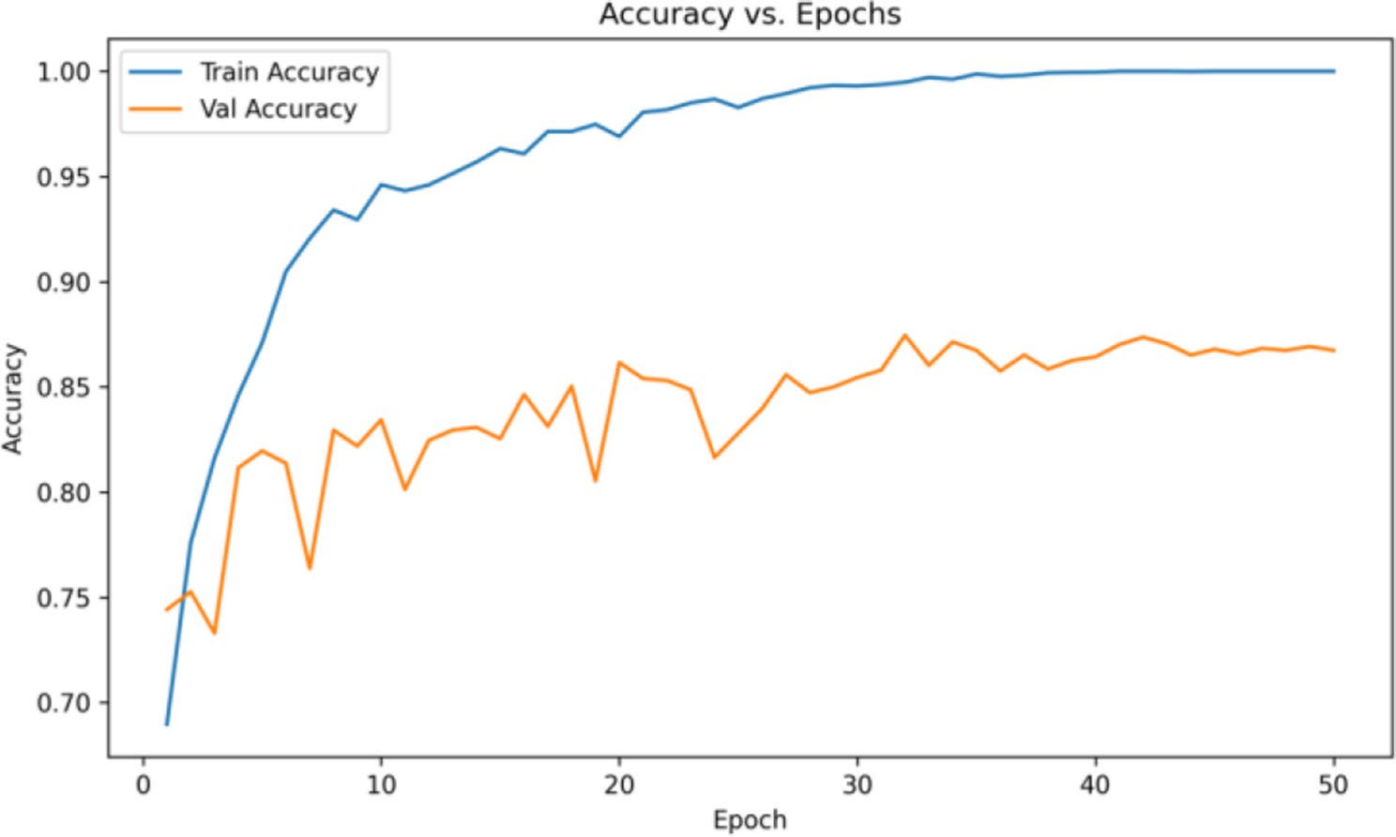
Accuracy curve per epoch for train set and validation set

**Fig. 11:**
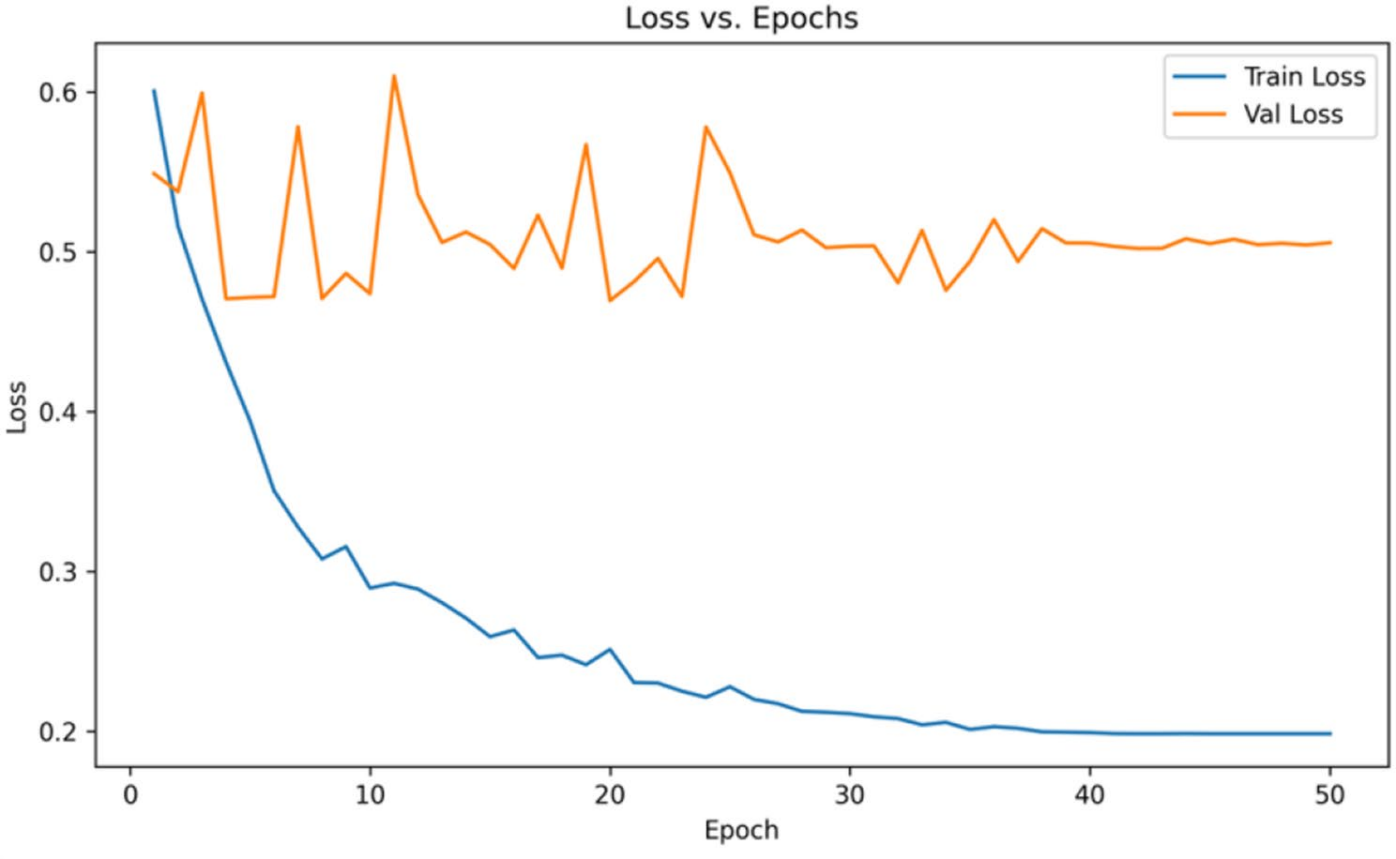
: loss curve per epoch for train set and validation set

The accuracy and loss values are tracked during the model training and validation phase as shown in Fig. 10 and Fig. 11. The training process achieved a final training loss of 0.20. The best validation performance was recorded at epoch 20, where the validation accuracy reached 86.16% and the validation loss was 0.4695. The model checkpoint saved at this point is used for evaluation on the test set.

**Fig. 12:**
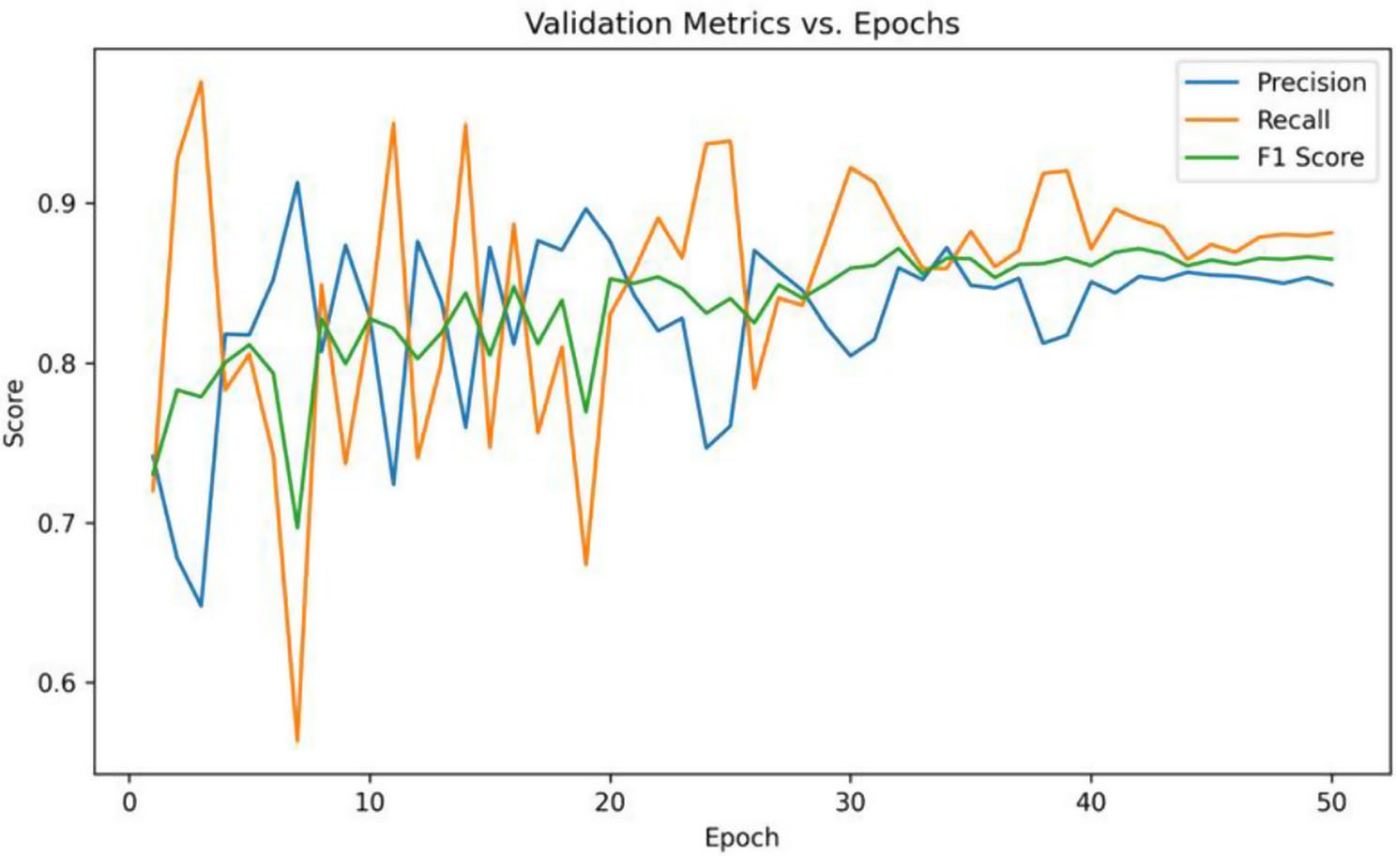
Precision, recall and F1 scores per epoch for validation set

The results as shown in Fig. 12 highlight the strengths and limitations of the model in terms of precision, recall and F1 score. While early fluctuations are visible, all three metrics converge to stable values of around 0.85. This reflects a balanced performance, which is crucial in medical diagnosis to minimize both false positives and false negatives.

**Fig. 13:**
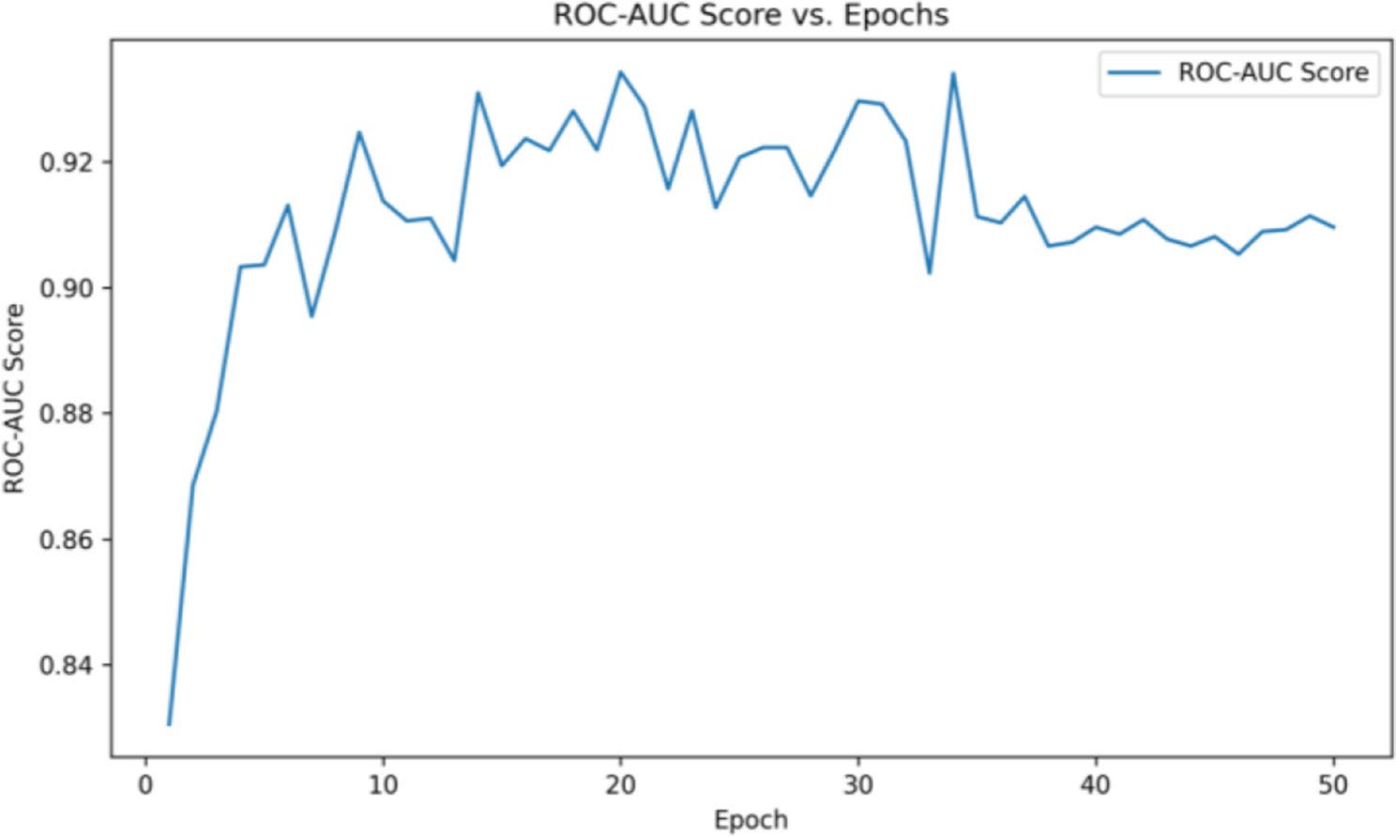
ROC-AUC score per epoch for validation set

Fig. 13 captures the model’s discriminative ability across epochs, showing how well the classifier separates TB-positive and TB-negative samples over time. The ROC-AUC quickly surpasses 0.90 in early epochs, indicating that the model rapidly learns to distinguish between the presence and absence of TB with high reliability.

### Comparison of test results

To investigate the performance of the proposed CoAtNet model, we benchmark it against several established architectures that represent key milestones in computer vision.

#### Baseline model architectures

- ResNet50: A foundational Convolutional Neural Network (CNN), ResNet50 [27] introduced the concept of residual learning. Its deep architecture is built from blocks containing “shortcut connections” that allow the gradient to flow more easily during training, effectively mitigating the vanishing gradient problem in very deep networks. It has been a standard baseline for numerous medical imaging tasks.
- EfficientNet (B0/B3): EfficientNet [28] represents an advancement in CNN design focused on resource efficiency. Instead of scaling network depth, width, or resolution independently, it uses a compound scaling method to uniformly scale all three dimensions. This approach optimizes the accuracy-to-parameter ratio, making it a powerful yet computationally efficient model for tasks like lung disease classification.
- Vision Transformer (ViT-B16): Departing from convolutional principles, the Vision Transformer [29] applies the self-attention mechanism, originally from natural language processing, directly to image classification. It processes an image by dividing it into fixed-size patches, linearly embedding them, and feeding them into a standard Transformer encoder. ViT excels at capturing global, long-range dependencies within an image, a contrast to the local feature extraction of CNNs.

#### Baseline model selection and comparison

This selection of baseline models was guided by architectural relevance, alignment with existing literature, and benchmark readiness. EfficientNet was chosen because it shares a key architectural component with CoAtNet: the MBConv block with squeeze excitation. This allows for a direct analysis of how CoAtNet’s hybrid design improves upon a strong CNN baseline with similar foundational blocks. The B0 and B3 variants were included to enable a fair comparison across different model scales. ResNet50 serves as a widely validated, traditional deep CNN baseline, while ViT-B16 offers a pure transformer-based approach, allowing us to contrast CoAtNet’s hybrid design against both ends of the architectural spectrum.

Furthermore, the selection aligns with recent related work that employed similar preprocessing pipelines. [5] evaluated both ResNet50 and EfficientNet on CXR datasets, and [17] used ViT-B16 in a hybrid model for TB classification, making the comparisons relevant to the current state of the field. Finally, all chosen models are fine-tuned from readily available pretrained ImageNet weights under identical training settings, ensuring a consistent and fair fine-tuning process.

It is important to note that our baseline models, particularly EfficientNet, were used in their default configurations to provide a clear and interpretable benchmark. While these baselines could be enhanced with additional components like attention mechanisms, proposed primary model, CoAtNet, already integrates such features. This methodological choice ensures that the observed performance improvement can be more directly attributed to the novel hybrid structure rather than to an enhancement that could be applied to any baseline.

The performance metrics, including accuracy, precision, recall, F1 score, and ROC-AUC, are computed on a held-out test set and summarized in Table 5. A comprehensive literature search was conducted to find other studies reporting classification performance on the IN-CXR dataset. As of this study, no other peer-reviewed publications with comparable benchmarks were identified. Therefore, the results presented in Table 5 serve as a novel baseline for future research utilizing this important public dataset.

**Table 5:** Model performance on test set

Model	Accuracy	Precision	Recall	F1 Score	ROC-AUC	Runtime
ResNet50	78.76	79.68	77.11	78.37	87.37	1 h 4 m
EfficientNetB0	80.68	77.88	85.61	81.56	89.24	**47 m**
EfficientNetB3	75.75	73.37	80.69	76.86	84.31	1 h 27 m
ViT B16	70.57	70.45	70.68	70.56	78.30	4 h 4 m
CoAtNet − 0	84.74	82.97	87.35	85.10	92.90	1 h 16 m
CoAtNet − 1	**86.39**	**89.24**	82.70	**85.85**	**93.79**	3 h 30 m
CoAtNet − 2	83.76	80.88	**88.33**	84.44	92.00	3 h 16 m

Among the models tested, CoAtNet-1 consistently outperforms the others, achieving the highest values in accuracy, precision, F1 score, and ROC-AUC. These metrics indicate that CoAtNet-1 classifies TB cases with high accuracy and maintains a strong balance between minimizing false positives and false negatives, making it suitable for clinical applications where diagnostic precision and reliability are critical. The precision of 0.8924 suggests very few false positives, reducing the likelihood of incorrect diagnoses, and the F1 score of 0.8585 reflects a balanced performance even on an imbalanced dataset. Additionally, the ROC-AUC of 0.9379 demonstrates strong discriminatory power and stable classification performance across thresholds.

To further scrutinize the proposed model’s performance, we compared it against SynthEnsemble [13], a powerful ensemble architecture that also includes CoAtNet as a component. While SynthEnsemble reported a strong average ROC-AUC of 85.43% on the ChestX-ray14 dataset, the proposed CoAtNet-1 model achieved a substantially higher ROC-AUC of 93.79% on the IN-CXR dataset in this study. This represents an absolute improvement of + 8.36%, showing that our model outperforms an ensemble approach on the given task. Although SynthEnsemble’s results are reported on a different dataset, their comparison with CoAtNet’s performance on IN-CXR remains informative given the similarity of the evaluation settings.

The results in Table 5 also highlight the critical trade-off between model complexity, runtime, and classification performance. The most lightweight model, EfficientNetB0, had the fastest runtime of just 47 minutes but achieved significantly lower accuracy and ROC-AUC scores. Conversely, the Vision Transformer (ViT-B16) had the longest runtime of over 4 hours but yielded the poorest performance, indicating that its architectural design was less suited for this task. The enhanced CoAtNet-1 strikes an effective balance. While its runtime of 3 hours and 30 minutes is not the lowest, it delivered the highest overall performance. This demonstrates that its hybrid architecture provides a superior balance of efficiency and clinical utility, justifying the moderate increase in computational cost for a significant gain in diagnostic reliability.

CoAtNet-2 stands out for its superior recall (0.8833), outperforming all other models in identifying true TB-positive cases. This makes it valuable in screening and early detection scenarios were identifying as many positive cases as possible is essential to prevent delayed diagnosis and reduce disease transmission. However, this higher recall comes with lower precision (0.8088), meaning more false positives and a greater need for additional confirmatory tests, increasing healthcare utilization. This illustrates a fundamental challenge in medical AI: balancing sensitivity (the ability to detect all positive cases) with specificity (the ability to minimize incorrect positive predictions). A model with high recall is important in scenarios where early detection is critical to patient outcomes and public health, while a model with higher precision is preferred in diagnostic workflows where false alarms can lead to avoidable interventions. Notably, CoAtNet-2s recall advantage over EfficientNetB0 (0.8833 vs. 0.8561) could correctly identify approximately 27 additional TB-positive patients per 1,000 true TB cases, enabling better treatment and disease diagnosis.

The CNN-based models, ResNet50 and EfficientNet, consistently underperform, demonstrating limitations in capturing complex spatial patterns in CXR images. ViT B16, while competitive with an accuracy of 70.57%, still falls short of the CoAtNet variants. This is likely due to the absence of convolutional inductive bias, a crucial feature in medical imaging tasks where local textures and structures play an essential role in accurate classification.

Thus, the CoAtNet architecture demonstrates superior performance in TB classification compared to the tested CNN and transformer baselines. CoAtNet-1 is the most balanced, making it ideal for general clinical applications, while CoAtNet-2 offers advantages in sensitivity-driven applications, particularly in early TB detection and screening. Additionally, the best result (CoAtNet-1, accuracy: 86.39%) on the enhanced CoAtNet model for TB classification showcases an improvement on the Top-1 ImageNet accuracy of the original CoAtNet-1 (83.3%) as reported in [2].

The ablation study in Table 6 highlights the contribution of each enhancement to the CoAtNet-1 model’s performance. Replacing AdamW with Adam led to a noticeable drop in precision (−5.86%), accuracy (−2.43%), and ROC-AUC (−2.00%), indicating weaker confidence in positive predictions and reduced overall discrimination. However, recall slightly increased by 2.06%. This precision-recall trade-off emphasizes AdamW’s strength in achieving better generalization through effective weight decay regularization. Removing label smoothing caused a decline in accuracy (−1.67%), F1 score (−1.21%), and ROC-AUC (−0.89%), and a clear drop in precision (−4.35%). This shows that label smoothing helps prevent the model from becoming overconfident, promoting better-calibrated and more generalizable predictions. Excluding the ReLU activation produced only minor changes: AUC dropped by 0.41%, and precision by 0.79%, while accuracy and F1 score remained nearly the same. This indicates that while ReLU contributes to sharper decision boundaries, the model retains strong performance due to its robust architecture. Overall, results validate the design choices, indicating that each component plays a key role in enhancing the model’s diagnostic performance.

**Table 6:** Ablation studies

Model Configuration	Accuracy	Precision	Recall	F1 Score	ROC-AUC
Full Model (CoAtNet-1)	86.39	89.24	82.70	85.85	93.79
Without AdamW (Uses Adam)	83.96	83.38	84.76	84.06	91.79
Without Label Smoothing	84.72	84.89	84.40	84.64	92.90
Without ReLU Activation	86.32	88.45	83.50	85.90	93.38

## Conclusion

In conclusion, the enhanced CoAtNet architecture has significantly advanced the detection of tuberculosis in CXR images, demonstrating improved performance over tested baseline models such as ResNet50, EfficientNet (B0 and B3), and the ViT model ViT-B16 on the IN-CXR dataset. To ensure robustness, the 5-fold cross-validation confirmed the consistency of performance across different data splits. The use of extensive data preprocessing techniques was instrumental in reducing overfitting and enhancing the model’s generalisation capabilities. Fine-tuning of model parameters, paired with the application of the AdamW optimiser and Cross-Entropy Loss with label smoothing, ensured optimal performance during training.

The model achieved an accuracy of 86.32% and an ROC-AUC score of 93.38%, highlighting its strong predictive capabilities on a diverse and representative dataset. The integration of LIME provided interpretability and transparency, making the model’s predictions understandable and trustworthy for clinicians. These results underscore the potential of the proposed architecture to deliver reliable and explainable AI-driven solutions for automated tuberculosis classification in medical imaging. It is important to note that these findings are based on a comprehensive internal validation using a single dataset. While the use of 5-fold cross-validation strengthens this work’s results, broader claims of clinical readiness or general superiority require further rigorous testing. Future work must focus on validating the model’s performance on external, independent datasets from different geographic locations and clinical settings, incorporating datasets from different demographic populations to further enhance generalizability and clinical applicability. Future research can also extend this framework to support multi-class classification, enabling differentiation between various pulmonary conditions beyond binary tuberculosis detection, and explore additional explainable AI (XAI) methods beyond LIME to strengthen the reliability and trustworthiness of computer-aided diagnostics.

From a clinical perspective, the model’s accuracy and interpretability can greatly improve diagnostic efficiency, reduce radiologist workload, and support early intervention in tuberculosis management.

## Data Availability

The dataset used and/or analyzed during the current study is provided by Indian Council of Medical Research (ICMR)-National Institute for Research in Tuberculosis (NIRT) and is available from the https://www.nirt.res.in/html/xray.html.
